# A multistrain approach to studying the mechanisms underlying compatibility in the interaction between *Biomphalaria glabrata* and *Schistosoma mansoni*

**DOI:** 10.1371/journal.pntd.0005398

**Published:** 2017-03-02

**Authors:** Richard Galinier, Emmanuel Roger, Yves Moné, David Duval, Anaïs Portet, Silvain Pinaud, Cristian Chaparro, Christoph Grunau, Clémence Genthon, Emeric Dubois, Anne Rognon, Nathalie Arancibia, Bernard Dejean, André Théron, Benjamin Gourbal, Guillaume Mitta

**Affiliations:** 1 Univ. Perpignan Via Domitia, IHPE UMR 5244, CNRS, IFREMER, Univ. Montpellier, Perpignan, France; 2 MGX-Montpellier GenomiX, Montpellier Genomics and Bioinformatics Facility, Montpellier, France; University of Cambridge, UNITED KINGDOM

## Abstract

In recent decades, numerous studies have sought to better understand the mechanisms underlying the compatibility between *Biomphalaria glabrata* and *Schistosoma mansoni*. The developments of comparative transcriptomics, comparative genomics, interactomics and more targeted approaches have enabled researchers to identify a series of candidate genes. However, no molecular comparative work has yet been performed on multiple populations displaying different levels of compatibility. Here, we seek to fill this gap in the literature. We focused on *B*. *glabrata* FREPs and *S*. *mansoni Sm*PoMucs, which were previously demonstrated to be involved in snail/schistosome compatibility. We studied the expression and polymorphisms of these factors in combinations of snail and schistosome isolates that display different levels of compatibility. We found that the polymorphism and expression levels of FREPs and *Sm*PoMucs could be linked to the compatibility level of *S*. *mansoni*. These data and our complementary results obtained by RNA-seq of samples from various snail strains indicate that the mechanism of compatibility is much more complex than previously thought, and that it is likely to be highly variable within and between populations. This complexity must be taken into account if we hope to identify the molecular pathways that are most likely to be good targets for strategies aimed at blocking transmission of the parasite through the snail intermediate host.

## Author summary

Schistosomiasis is the second most widespread human tropical parasitic disease after malaria. It is caused by flatworms of the genus *Schistosoma*, and poses a considerable threat for human health in numerous Asian, African and South American countries. The World Health Organization has set the goal of eradicating schistosomiasis by 2025. However, no vaccine is available, and we currently have only one drug (praziquantel) that can effectively and efficiently treat the disease. As treatment by mass drug administration would enhance the risk of drug resistance in schistosome parasites, complementary strategies to fight this parasitic disease are urgently needed. Freshwater snails of the *Biomphalaria* genus act as intermediate hosts in the transmission of the schistosome species. Thus, learning more about the mechanisms of the interaction between these snails and the schistosomes could critically facilitate the identification of potential new candidate molecules that may be targeted to prevent schistosome transmission in the field.

## Introduction

Schistosomes are the causative agents of schistosomiasis, which is one of the most important neglected human tropical diseases in the world. Schistosomes infect over 200 million people worldwide, causing both acute and chronic debilitating diseases [1,2]. There is no effective vaccine against schistosomes, and the treatment of schistosomiasis still relies on a single drug: praziquantel [3]. Praziquantel resistance can be easily selected experimentally [4], and some human populations subjected to mass treatment now show evidence of reduced drug susceptibility [5]. Thus, we need alternate control strategies. Toward this end, researchers have sought to block disease transmission at the level of the snail that acts as the intermediate host. However, if we hope to identify target genes that may be used to develop new strategies aimed at disrupting the transmission of schistosomiasis, we must decipher the mechanisms through which snails and schistosomes interact. Over the past four decades, numerous investigators have sought to understand these mechanisms by focusing on the interaction between *Biomphalaria glabrata* and *Schistosoma mansoni*, which was chosen as a model system.

The genetic determinism of the compatibility between *B*. *glabrata* and *S*. *mansoni* was clearly demonstrated by the C.S. Richards group in the 1970s [6,7]. Since then, several research groups have investigated the underlying molecular determinants using different laboratory strains of snails and schistosomes. Genetic studies of crosses between snail lines displaying compatible and incompatible phenotypes have revealed some candidate loci, including a gene cluster containing a super oxide dismutase (SOD)-encoding gene [8–10] and a genomic region containing genes putatively involved in parasite recognition [11]. Various transcriptomic comparisons have also been performed on other compatible and incompatible strains of snails and schistosomes [12–16]. These studies uncovered a series of candidate genes involved in recognition, effector, and signaling pathways that could contribute to the compatibility process (see [17] for a recent review). Taken together, the previous reports clearly show that the success or failure of *S*. *mansoni* in infecting *B*. *glabrata* reflects a complex interplay between the host’s defense mechanisms and the parasite’s infective strategies. Little is known about the molecular variability playing of these molecular determinants underlying the compatibility; only one work has studied and shown the differential allelic expression of a SOD gene in different individuals of the predominantly resistant 13-16-R1 strain of *B*. *glabrata* [10]. The objective of the present work is to fill this gap by studying the molecular determinants of compatibility in different populations with varied compatibility phenotypes, in order to evaluate potential between-population differences in the compatibility mechanisms. To achieve this aim, we focused on molecular determinants known to be involved in snail/schistosome compatibility, and studied their expressions and polymorphisms in host and parasite isolates that differ in their compatibilities. We first studied the *Sm*PoMucs (polymorphic mucins from *S*. *mansoni*), which were initially identified by a comparative proteomic analysis of two strains of *S*. *mansoni* that differed in their compatibility towards the same mollusk strain [18]. *Sm*PoMucs share the general features of mucins, including a N-terminal domain containing a variable number of tandem repeats and a conserved C-terminal domain [19]. These proteins are expressed only by larval-stage parasites during interactions with the snail intermediate host; they are produced and located in the apical gland of miracidia and sporocysts, and are characterized by high levels of glycosylation and polymorphism [19,20]. A detailed analysis of intra- and inter-strain *Sm*PoMuc polymorphisms revealed that the diversification of these proteins has been driven by a complex cascade of mechanisms involving recombination between genes of the multigene *Sm*PoMuc family (10 genes), epigenetic control of transcription, post-transcriptional regulation events, and post-translational modifications [20–22]. This yields a remarkably high degree of diversification from a limited set of genes, enabling each individual parasite to express a specific and unique pattern of *Sm*PoMucs [20]. Functionally, these proteins are thought to play roles in the very early steps of infection [23]. Based on the above findings, it has been proposed that *Sm*PoMucs could be crucial antigens in the snail-schistosome compatibility process. Then, we developed co-immunoprecipitation (CoIP) experiments that enabled us to identify putative *Sm*PoMuc-interacting immune receptors of the snail [24]. We found that *Sm*PoMucs form molecular complexes with the fibrinogen-related proteins (FREPs) of *B*. *glabrata* [25]. FREPs are highly polymorphic, with somatic diversification generating unique repertoires in individual *B*. *glabrata* [26]. Thus, we considered these proteins to be good candidates as molecular determinants on the snail side of the compatibility between *B*. *glabrata* and *S*. *mansoni*. This importance of FREPs in the compatibility process was confirmed by specific knockdown of FREP 3 in *B*. *glabrata* BS-90 snails, which are totally resistant to a specific laboratory strain of *S*. *mansoni* [27]. The knockdown snails lost 21.4% of their resistance to *S*. *mansoni* infection, suggesting that FREP 3 participates in recognition but is not the sole determinant.

As FREP immune receptors and their *Sm*PoMuc antigens are clearly involved in the compatibility process, we herein focused on these molecular determinants of the recognition process between the host and the parasite.

We first characterized the compatibilities among all sympatric and allopatric combinations of four strains of *S*. *mansoni* (two from Brazil, one from Venezuela, and one from Guadeloupe Island) and four strains of *B*. *glabrata* (from the same locations) from South America and the Caribbean area. We then used targeted approach to analyze the expressions of *Sm*PoMucs in Schistosomes and global transcriptomic approach on FREPs between the four strains of *B*. *glabrata*. The global transcriptomic analysis of snail strains also revealed large transcriptional differences, especially for the *B*. *glabrata* strain that showed the least compatibility when confronted with the studied schistosome strains. Global transcriptomic differences were observed among numerous genes involved in the different phases of the immune response. Based on our findings, we propose that the compatibility between *B*. *glabrata* and *S*. *mansoni* depends on a multistep process that involves both recognition and effector/anti-effectors systems.

## Results

### A multistrain approach for assessing compatibility phenotypes

As the objective of the present work was to evaluate the putative link between the expression patterns of *Sm*PoMucs and FREPs and the compatibility between snails and schistosomes, we first established the compatibility levels of four strains of *B*. *glabrata* (*Bg*BAR, *Bg*VEN, *Bg*BRE, and *Bg*GUA) when confronted with four strains of *S*. *mansoni* (*Sm*LE, *Sm*VEN, *Sm*BRE, and *Sm*GH2). Compatibility was tested for all sympatric and allopatric combinations.

**Fig 1:**
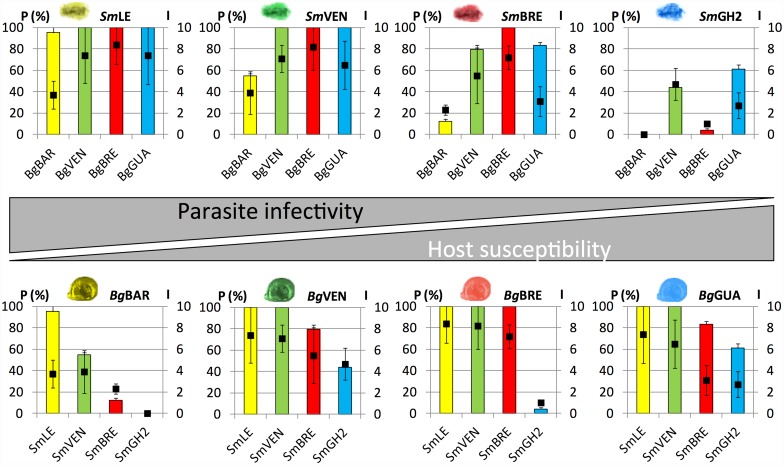
Compatibility trials between different strains of parasites and snails. Each pairwise combination of the studied strains of schistosomes (*Sm*LE, *Sm*VEN, *Sm*BRE, and *Sm*GH2) and snails (*Bg*BAR, *Bg*VEN, *Bg*BRE, and *Bg*GUA) were tested for compatibility. The upper graphs present the prevalence (P in %) and intensity (I) of infection for each *S*. *mansoni* in the different *B*. *glabrata* host strains. The lower graphs present the prevalence and intensity of infection for each *B*. *glabrata* strain when confronted by the four *S*. *mansoni* strains. Prevalence values are represented by colored histograms, while intensity values are indicated by black squares. The sympatric snails and schistosomes bear the same color. The presented data represent the mean values obtained for three independent experiments, the data obtained by (Theron et al, 2014) and two other experiments performed in 2010 and 2012. Error bars represent the average absolute deviation of the mean.

All strain combinations displayed different compatibility levels (Fig 1). *Sm*LE displayed the highest compatibility, showing a 96–100% prevalence of infection when confronted with the four *B*. *glabrata* strains. *Sm*GH2 showed the least compatibility, exhibiting prevalences of 0–44% for allopatric combinations and only 60% when confronted with its sympatric mollusk. *Sm*VEN was highly compatible (100%) with its sympatric mollusk strain and with *Bg*BRE and *Bg*GUA, but it was less efficient when infecting the *Bg*BAR snail strain (55%). *Sm*BRE displayed an intermediate compatibility phenotype; it showed 100% prevalence for its sympatric mollusk *Bg*BRE, and its compatibility ranged from 12% to 83% when confronted by the other mollusk strains. Our results for the intensity of infection followed similar trends, ranging from 1 to 8.4 parasites per infected snails (Fig 1). The infective capacities of the four parasite strains exhibited a gradient of compatibility in the following descending order: *Sm*LE, *Sm*VEN, *Sm*BRE, and *Sm*GH2.

Considering the mollusk strains, *Bg*BAR was the least compatible; it exhibited prevalences of 0–55% when confronted with allopatric parasite strains and reached 96% when infected with its sympatric *Sm*LE, but the intensity of infection never exceed 3.9 (even for *Sm*LE). *Bg*VEN, *Bg*BRE, and *Bg*GUA showed very similar compatibility patterns, exhibiting complete compatibility (prevalence, 100%) when exposed to *Sm*LE and *Sm*VEN, but less compatibility with *Sm*GH2. *Bg*GUA and *Bg*VEN displayed very similar compatibility patterns, showing slightly more infectivity when confronted with *Sm*BRE than *Sm*GH2. *Bg*BRE was totally compatible with its sympatric schistosome, but displayed a very low compatibility with *Sm*GH2 (4%). The intensities were generally high, largely between 4.7 and 8.4. Our results indicate that *Bg*BAR is the least compatible host strain, whereas *Bg*VEN, *Bg*BRE, and *Bg*GUA are much more compatible and display quite similar compatibility phenotypes.

### SmPoMuc polymorphism analysis

**Fig 2:**
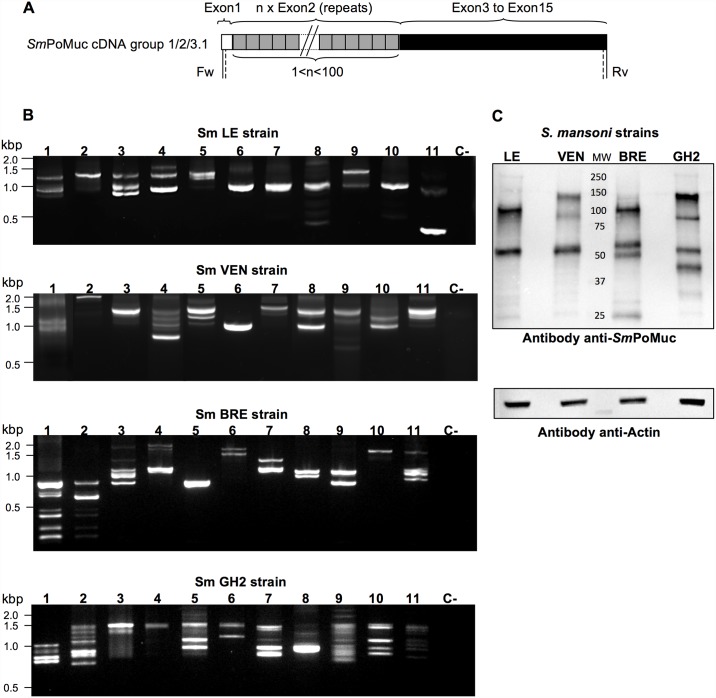
Diverse *Sm*PoMucs are expressed by the different strains of *S*. *mansoni*. (A) *Sm*PoMuc cDNA structure. The 5’ region is characterized by a variable number of tandem repeats. Two different types of repeats (r1 and r2) were mainly identified in previous studies [19,20]. SmPoMuc cDNA structure is shared by previously published *Sm*BRE, and *Sm*GH2 strains [19,20] and the new ones, *Sm*LE and *Sm*VEN. The 3’ region (exon 3 to exon 15) harbors sequence differences that enable the proteins to be classified into the only three groups (groups 1, 2, and 3.1) known to be expressed [19,20]. (B) Transcription patterns of *Sm*PoMucs in 11 individuals of each schistosome strain. Nested RT-PCR amplicons were obtained from individual sporocysts (1 to 11) of *Sm*LE, *Sm*VEN, *Sm*BRE, and *Sm*GH2, and resolved by agarose gel electrophoresis. PCR was performed using consensus primers that amplified the complete coding sequence of all *Sm*PoMuc transcripts (located in exon 1 and exon 15; vertical and dashed lines represent the positions of the primers used for PCR and nested PCR, respectively). “C-” denotes the negative control. *Sm*BRE and *Sm*GH2 nested PCR results were from [21] (C) Inter-strain polymorphism at the protein level. Protein extracts (8 μg) were obtained from sporocysts of each *S*. *mansoni* strain, and Western blot analysis was performed using anti-*Sm*PoMuc polyclonal antibodies. Actin was detected as a control.

We previously showed that the *Sm*PoMucs are encoded by a multi-gene family of 10 members that can be divided into four paralogous sequence groups (groups 1–4) [20]. Fig 2A shows the *Sm*PoMuc cDNA structure shared by the different groups. Their 5’ regions comprise a variable number of tandem repeats corresponding to repetitions of exon 2, whereas the 3’ regions (exons 3 to 15) differ in their sequences, enabling the *Sm*PoMucs to be divided into groups 1 through 4. At the transcript level, only groups 1, 2 and 3.1 have been detected to date [20]. *Sm*PoMucs of groups 1 and 2 contain the same type of exon 2 (r2, 27 nucleotides) that is repeated in the transcript structure, while those of group 3 contain a different type of repeated exon 2. Most group 3 SmPoMucs have r1 as their repeated sequence, except for the subgroup denoted group 3.1 (r1-r2), which displays both types of exon 2 (r1 and r2) in the gene structure. These recombined genes are present in the genomes of different schistosome strains, but appear to be expressed only in *Sm*GH2 (see also [19,20]).

To analyze *Sm*PoMuc transcript polymorphism, RNA was extracted from 11 individual sporocysts of each strain (*Sm*BRE, *Sm*VEN, *Sm*LE, and *Sm*GH2) and subjected to nested RT-PCR. Consensus primers (see Fig 2A for the positions of the utilized primers) were used to amplify the complete coding sequence of all *Sm*PoMucs in each individual. Fig 2B shows the *Sm*PoMuc banding patterns obtained on agarose gels.

We first examined inter-strain variability. Consistent with the results of previous studies performed using the same method [20], the *Sm*PoMuc banding patterns were highly different across the analyzed strains. To analyze this polymorphism at the protein level, we analyzed *Sm*PoMucs by Western blotting. Proteins from 5000 individuals of each *S*. *mansoni* strain were resolved and detected with an anti-*Sm*PoMuc antibody directed against a conserved region of the *Sm*PoMucs [24]. As shown in Fig 2C, the *Sm*PoMuc protein patterns differed across *Sm*LE, *Sm*VEN, *Sm*BRE, and *Sm*GH2, each isolate expressed a specific *Sm*PoMuc profile.

With respect to intra-strain comparisons, the polymorphism was found to be very high at the transcript level. The *Sm*BRE and *Sm*GH2 banding patterns were obtained from a previous work [21] and the banding patterns of *Sm*VEN and *Sm*LE was obtained in the present work. For the 4 *S*. *mansoni* isolates, no two individuals display the same amplification profile (Fig 2B). To more precisely characterize these patterns, we sequenced the amplicons obtained from each individual of the four *S*. *mansoni* strains. The results are shown in S1 Table. All individuals expressed multiple variants; some expressed only variants belonging to a single group of *Sm*PoMucs, while others expressed variants from two or three groups. Within each group, the *Sm*PoMuc transcript polymorphisms reflected: (i) a variable number of tandem repeats (from 1 to 100) in the 5’ region; (ii) the occurrence of alternative and aberrant splicing in the 3’ region; and (iii) nucleic acid substitutions (synonymous or non-synonymous) in the 5’ and 3’ regions. These different mechanisms were previously reported [19,20]. We did not observe any clear link between the presence of a given variant and the compatibility level of the different strains, with one exception. A sub-group of variants belonging to *Sm*PoMuc group 3.1, named group 3.1(r1-r2), was more abundant in the less compatible *Sm*GH2 strain than in the other strains. This variant was found in six individuals of *Sm*GH2, but in only one individual each of *Sm*BRE and *Sm*VEN and no individual of *Sm*LE.

**Fig 3:**
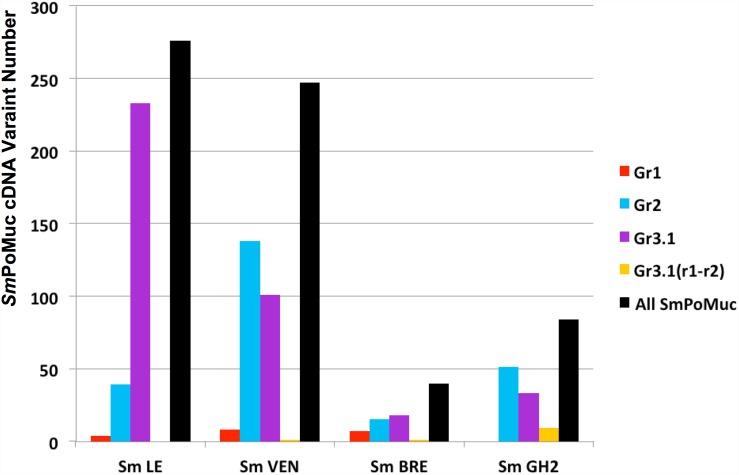
*Sm*PoMuc variant numbers in the different *S*. *mansoni* strains. Nested RT-PCR for *Sm*PoMucs was performed on 11 individual sporocysts for each *S*. *mansoni* strain (*Sm*LE, *Sm*VEN, *Sm*BRE, and *Sm*GH2), and the obtained fragments were cloned and analyzed by Sanger sequencing (see S1 Table). The numbers of *Sm*PoMuc cDNA variants obtained for group 1 (red), group 2 (blue), group 3.1 (purple), group 3.1(r1-r2) (yellow), and all groups (black) are indicated.

Interestingly, *Sm*LE and *Sm*VEN, which were the most infective strains, displayed a larger number of *Sm*PoMuc variants (276 and 247, respectively) than the less infective *Sm*BRE and *Sm*GH2 strains (40 and 84, respectively). It is interesting to note that all of the 647 variants sequenced in the present study were different. These results are shown in Fig 3.

### SmPoMucs are differentially expressed between *S*. *mansoni* strains

**Fig 4:**
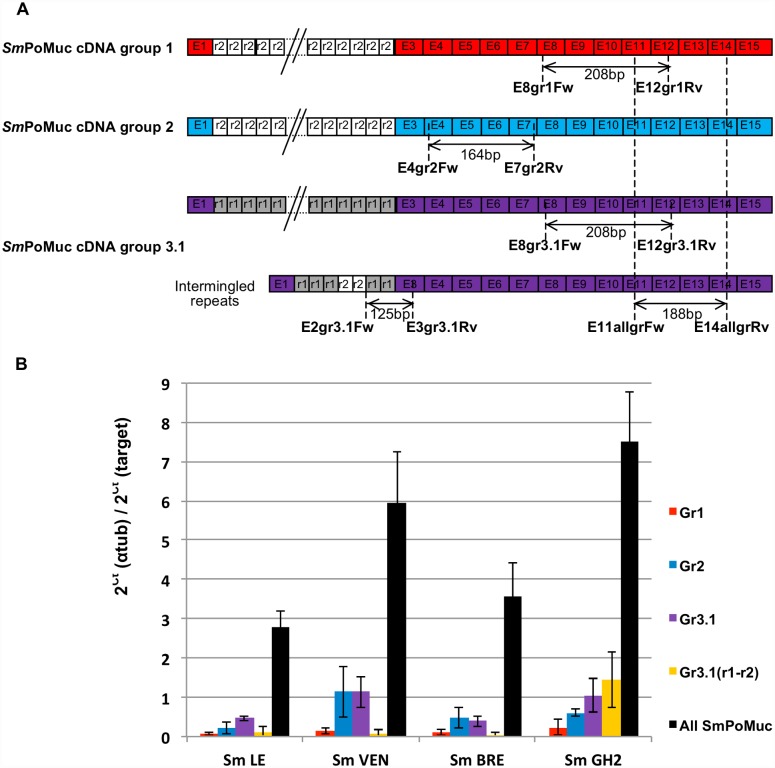
*Sm*PoMuc expression between *S*. *mansoni* strains. (A) Quantitative PCR was performed with primers that targeted all *Sm*PoMuc transcripts or discriminated among groups 1, 2, 3.1, and 3.1(r1-r2). The primer positions and amplicon sizes are indicated. (B) RNA was extracted from pooled sporocysts of each *S*. *mansoni* strain (*Sm*LE, *Sm*VEN, *Sm*BRE, and *Sm*GH2). The results are presented as the mean Ct values normalized with respect to the α-tubulin gene, and were obtained from three biological replicates.

The expression levels of *Sm*PoMuc transcripts were assessed for each *S*. *mansoni* strain by RT-Q-PCR. Primers E11allgrFw and E14allgrRv were universal to all *Sm*PoMuc genes, whereas the other utilized primers (see Fig 4) allowed us to quantify the transcripts corresponding to the group 1, group 2, group 3.1, and group 3.1 (r1-r2) *Sm*PoMuc genes. The positions of the utilized primers are indicated in Fig 4A. Relative expression ratios were calculated using α-tubulin as a reference gene (Fig 4B).

Our results revealed that the levels of *Sm*PoMuc transcripts of all groups differed across the four schistosome strains. *Sm*LE and *Sm*BRE displayed the lowest levels of *Sm*PoMuc transcripts, and *Sm*VEN and *Sm*GH2 expressed around 2 and 2.5 fold more *Sm*PoMuc transcripts, respectively (Mann-Whitney test: LE vs VEN P = 0.0033; LE vs GH2 P = 0.0079; BRE vs VEN P = 0.0021; BRE vs GH2 P = 0.0025). With respect to the different *Sm*PoMuc groups, the transcription patterns were quite similar between the strains: group 1 was weakly expressed in all cases, whereas group 2 and 3.1 transcripts were expressed at higher levels (2- to 15 fold more, depending on the group and strain). The group 3.1 (r1-r2) *Sm*PoMuc transcripts were highly expressed only in strain *Sm*GH2 (Mann-Whitney test: GH2 vs LE P = 0.0079; GH2 vs BRE P = 0.0025; GH2 vs VEN P = 0.0009).

Interestingly, the sum of the group-level expression data did not correspond to the *Sm*PoMuc expression revealed by the *Sm*PoMuc universal primers. This may reflect that some of the spliced variants of *Sm*PoMucs revealed in a previous study [20] are amplified only with universal primers. As no specific primer could be designed to specifically amplify these spliced variants, we were unable to test this hypothesis.

### RNA-seq-based analysis of the four *B*. *glabrata* strains

**Fig 5:**
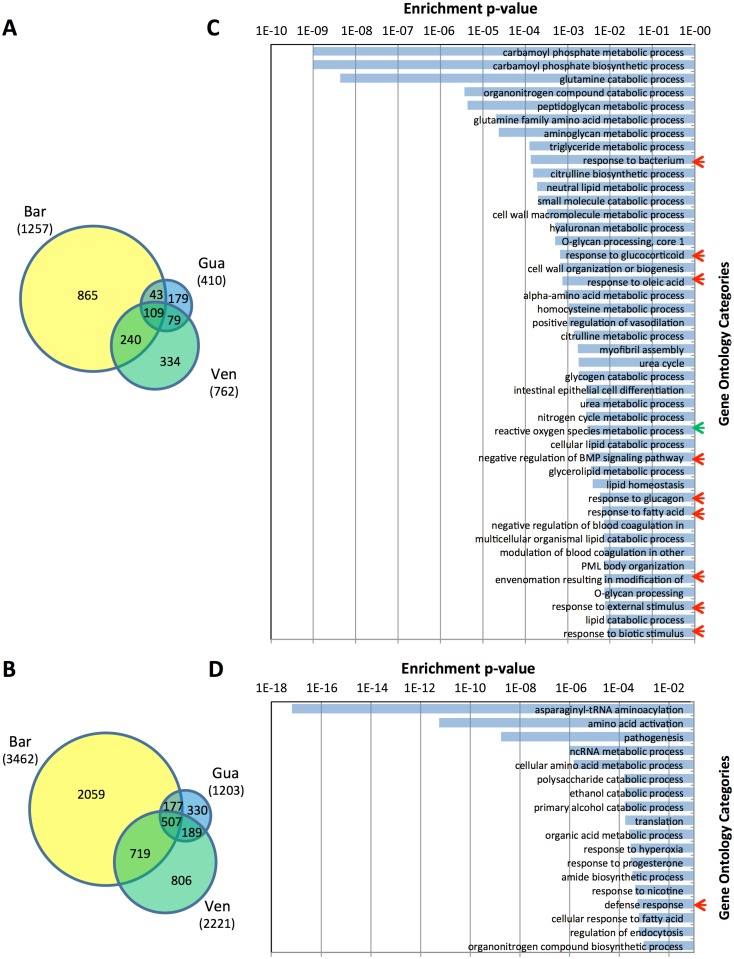
RNA-seq of the different *B*. *glabrata* strains, and enrichment analysis. Venn diagram presenting the transcripts found to be over-represented (A) and under-represented (B) in the transcriptomes of *Bg*BAR, *Bg*VEN, and *Bg*GUA versus *Bg*BRE. Gene Ontology (GO) enrichment analysis of genes that were over-represented (C) and under-represented (D) in the transcriptome of *Bg*BAR. Red arrows indicate biological processes belonging to the “response to biotic and abiotic stress” group, while the green arrow shows an immune-response-related biological process.

Until now, most of the experiments done on compatibility between Schistosomes and *Biomphalaria* snails were conducted using targeted Quantitative PCR or micro-array approaches to identified differentially represented transcripts following infection. In the present paper a more global and powerful approach was conducted to identify the differentially regulated transcripts or differential level of constitutive expression between snail strains. This global approach will also ensure a gene discovery effort without foreseeing the molecules involved compared to targeted approaches. To investigate such differences, four *B*. *glabrata* strains were used. The global transcript representation was analyzed by RNAseq and correlated with their compatibility phenotypes. *Bg*BAR was the less compatible strain, while *Bg*BRE, *Bg*VEN and *Bg*GUA present higher compatible phenotypes that were mostly similar (Fig 1). To compare the native immune potentials of the four *Bg* strains, we compared the biological replicates, *Bg*BRE1 and *Bg*BRE2, to the other *Bg* strains. *BgBRE* strain was selected for duplicate sequencing because it is the strain for which we have the largest number of individuals in the laboratory. To identify the differentially represented transcripts in the transcriptomes of the different strains, we used the DEseq2 software comparing the duplicate of *BgBRE* strain to the others transcriptomes. Of the 117,269 transcripts of the *Bg*BRE transcriptome, 6555 (5.6%) were found to be differentially expressed between *Bg*BRE and the three other *B*. *glabrata* strains. Fig 5A and 5B present the numbers of *Bg*BAR, *Bg*VEN, and *Bg*GUA transcripts that were significantly over- and under-represented, respectively. *Bg*BAR showed the highest number of differentially expressed transcripts (1257 over-represented and 3462 under-represented). In contrast, *Bg*VEN and *Bg*GUA showed differential expression (both over- and under-representation) of 2983 and 1613 transcripts, respectively. Of the 6555 modulated transcripts identified in the present study, 72%, 45%, and 25% were from *Bg*BAR, *Bg*VEN, and *Bg*GUA, respectively.

Based on our observations that *Bg*BAR differed the most from the other strains in both its molecular phenotype and its compatibility phenotype, we decided to analyze the putative functions that were enriched among its differentially expressed genes. We carried out a Gene Ontology (GO) enrichment analysis on the differentially expressed transcripts that were uniquely over-represented (865 transcripts, Fig 5C) and under-represented (2059 transcripts, Fig 5D) in *Bg*BAR relative to *Bg*BRE. The over-represented transcripts of *Bg*BAR corresponded to 85 biological processes representing 44 categories. Interestingly, eight categories (red arrows in Fig 5C) that contained 15% of the transcripts were grouped together by the REVIGO software into the biological process of “response to biotic and abiotic stress.” One additional category (3% of the transcripts) corresponded to the biological process of “reactive oxygen species metabolism.” These two biological processes are clearly of interest in our study context. Indeed reactive oxygen species (ROS) have been widely shown to be involved in snail/parasite compatibility [8,28,29]. Reactive oxygen species (ROS) produced by the hemocytes of *B*. *glabrata* are known to play a crucial role in killing *S*. *mansoni* [30,31]. This process could be related to the level of H2O2 expressed by resistant snails associated with a higher expression of SOD genes (copper/zinc superoxide dismutase) [8,32].

The under-represented transcripts of *Bg*BAR fell into 18 categories. Among them, only the “defense response” (3% of the transcripts) seems likely to be related to immune function.

**Fig 6:**
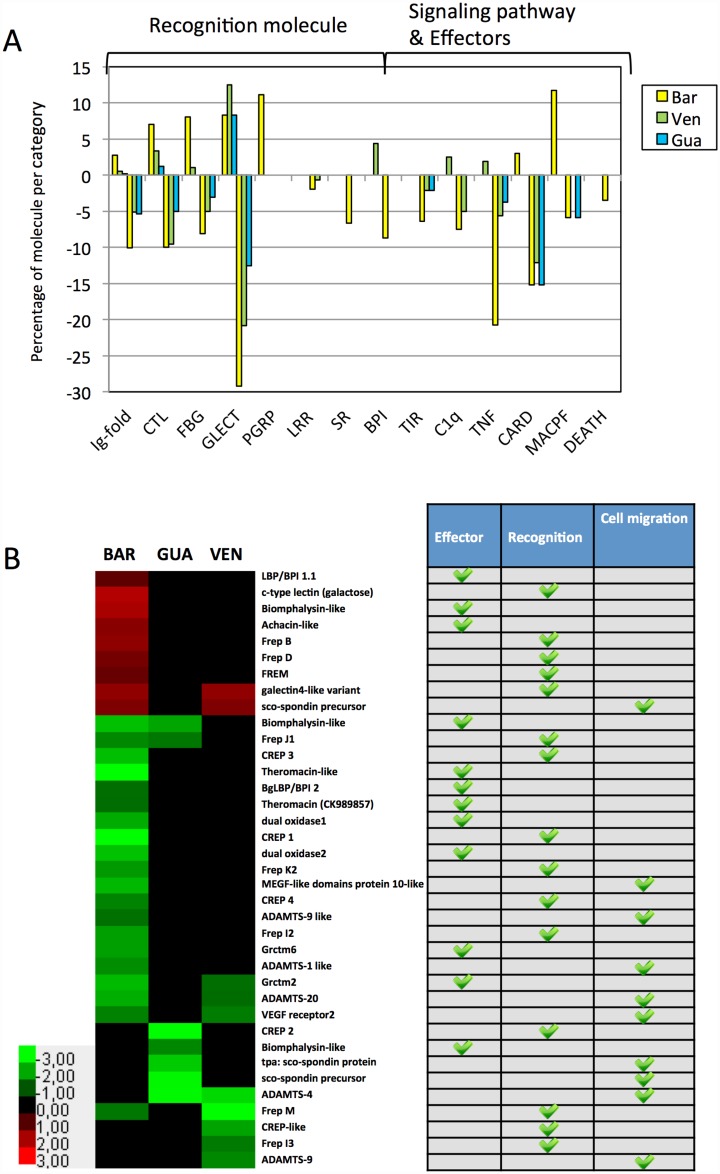
Analysis of immune-relevant genes differentially represented between *Bg*BAR, *Bg*VEN, *Bg*GUA and *Bg*BRE reference transcriptome. (A) Histograms showing the proportion of genes bearing immune-relevant protein domains that were differentially represented (DESeq 2 analysis) in *Bg*BAR (yellow), *Bg*VEN (green) and *Bg*GUA (blue) versus *Bg*BRE. The monitored immune-relevant genes include immune recognition molecules, immune signaling proteins, and immune effectors. Within these categories, the genes were subdivided by shared protein domains (defined by Interproscan analysis of predicted protein sequences). Ig-fold: immunoglobulin-like fold, IPR013783. CTL: C-type lectin, IPR001304. FBG: fibrinogen-related domains, IPR002181. GLECT: galectin, IPR001079. PGRP: animal peptidoglycan protein homolog, IPR006619. LRR: leucine-rich repeat, IPR001611. SR: scavenger receptor cysteine-rich, IPR017448. BPI: lipopolysaccharide-binding protein, IPR001124 and IPR017942. TIR: toll-interleukin 1-receptor, IPR000157. C1q: complement component C1q domain, IPR001073. TNF: tumor necrosis factor family, IPR006052. CARD: caspase recruitment domain, IPR001315. MACPF: membrane-attack complex/perforin, IPR020864. DEATH: DEATH domain, found in proteins involved in cell death, IPR000488. (B) Genes previously described to have an immune function in *B*. *glabrata* species. The heat map represents the log2 fold change values for transcripts that are differentially represented between the snail strains. The table beside the heat map classifies the corresponding genes into three immune functions: effector, recognition, and cell migration.

Considering the previous results presented above, we decided to deepen our investigation of immune functions in the different *Bg* strains. We first identified genes bearing immune-relevant domains and examined their proportions in the studied strains. We referred to a previous study in which the whole transcriptome of *Bg*BRE was screened for the presence of immune-relevant domains using Interproscan [33]. Using these data, we determined the proportion of the relevant transcripts that were differentially expressed in the strains studied herein. The results are shown in Fig 6A. Given the focus of the present paper and our interest in FREPs, we found it interesting that 3% and 10% of all transcripts bearing an “Ig-fold” domain were over and under-represented, respectively, in *Bg*BAR. This particular domain is shared by a family of variable immunoglobulin and lectin domain-containing proteins [33]; notably, this family includes numerous FREPs. A large proportion of the transcripts corresponding to proteins containing other recognition domains, as well as immune signaling molecules and effectors, were also differentially represented between *Bg*BRE and the other three strains. As expected, *Bg*BAR had higher proportions of differentially expressed genes for most of the categories of putative immune-relevant genes.

To study the immune function of the different snail strains more deeply, we constituted a *B*. *glabrata* immunome comprising 122 transcripts identified after immune challenge of *B*. *glabrata* or by comparative "omic" analyses of snail strains displaying different compatibility phenotypes towards trematodes (S2 Table) [11,17,34]. When we examined these immune-relevant molecules in *Bg*BAR, *Bg*GUA, and *Bg*VEN versus *Bg*BRE (Fig 6B), we identified 37 differentially expressed transcripts. Nine of them were over-represented and 28 were under-represented: of the latter group, 14 were specifically under-represented in *Bg*BAR. Again, *Bg*BAR is the most different among the strains; the nine over-represented transcripts displayed their highest representation in *Bg*BAR, while 20 of the 28 under-represented genes showed their lowest expression levels in this strain. The nine over-represented immune-relevant transcripts included the following: two FREP-encoding transcripts; a transcript containing a Aerolysin domain belonging to the epsilon toxin ETX/Bacillus mosquitocidal toxin MTX2 superfamily previously characterized as a biomphalysin-like protein shown to kill *S*. *mansoni* sporocysts [35]; and a transcript encoding an achacin-like, which suggests that *Bg*BAR may have an enhanced ability to respond to antimicrobial stress [34,36]. The under-represented transcripts included the following: several transcripts corresponding to FREP family members; transcripts encoding different molecules of the CREP family, whose members are composed of immunoglobulin domain(s) followed by a C-type lectin domain [33]; and transcripts encoding several factors involved in extracellular matrix remodeling and cell migration (e.g., sco-spondin, ADAMTS, and VEGF receptor).

### Characterization and expression analysis of FREPs in the four *B*. *glabrata* strains

As several FREPs (available in GenBank databases) were revealed in the above described transcriptomic analysis, we decided to exhaustively study all transcripts in the transcriptomes of *Bg*BAR, *Bg*VEN, *Bg*BRE, and *Bg*GUA that could belong to FREP family members. We selected transcripts corresponding to proteins that contain immunoglobulin (IPR013783) and fibrinogen (IPR002181) domains using the Blast2Go and/or Interproscan software packages. This selection process yielded 258 transcripts. Most of them were not full-length, which meant they could potentially represent molecules that contain fibrinogen or immunoglobulin domains but do not follow the classical organization of FREPs [1 or 2 IgSF domain(s) associate with a fibrinogen domain]. As our objective was to study FREPs-encoding transcripts, we retained all transcripts that contained contiguous immunoglobulin superfamily (IgSF) and fibrinogen (FBG) domains (full-length or partial). In total, we retained 69 FREP transcripts.

The 69 retained transcripts were translated, and the corresponding amino acid sequences were aligned with the *Bg* FREP protein sequences contained within GenBank. None was an exact match, but 30 of the 69 transcripts displayed only a few differences, and could thus be clearly assigned according to the GenBank FREP nomenclature [37]. The 38 remaining sequences could not be assigned to the established nomenclature. Next, we aligned the 69 sequences with the *B*. *glabrata* genome draft (www.vectorbase.org/organisms/biomphalaria-glabrata; genome assembly version BglaB1) and assigned them to precise positions in the genome (see supplementary S3 Table). The 69 transcripts corresponded to 24 different genomic loci. Several *de novo* assembled transcripts were validated by traditional Sanger sequencing of PCR products to confirm that this FREPs were not the result of wrong assemblies (S1 Fig). For this study, we classified FREPs by their identity and assigned genomic locus. Any two FREPs that shared more than 85% identity were considered as homologous and were given the same letter (designating FREP class); however if they occupied different genomic loci, the letter is followed by a different number (e.g., C1 and C2). We identified seven loci that encode FREPs with one IgSF domain (grouped into classes A to F), and 16 loci corresponding to FREPs with two IgSF domains (grouped into classes H to M). Finally *B*. *glabrata* genome assembly did not allow us to establish the number of IgSF domains for a last FREP class named “O”.

**Fig 7:**
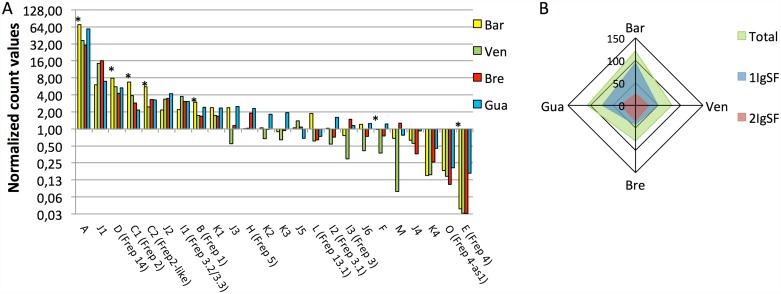
Abundances of transcripts for the different FREP classes in the four *B*. *glabrata* strains. (A) The histograms represent normalized count values (upper quartile method) obtained when Bowtie 2 was used to map the *Bg*BAR, *Bg*VEN, *Bg*BRE, and *Bg*GUA reads with respect to the sequences of the 24 classes of FREP transcripts. Asterisks indicate FREP classes that contain only one IgSF domain. (B) The diagram shows the sum of normalized count values for all the FREPs (total) or the FREPs containing one or two IgSF domains.

After we characterized these 24 loci of FREPs, we used RNA-seq to analyze the representation of the corresponding transcripts in the four *B*. *glabrata* strains. We built a FREP transcriptome containing the 69 selected sequences, mapped the reads obtained from each snail strain to these sequences using the Bowtie2 software, and normalized the hit count values for each transcript using the upper quartile method. The normalized hit count values were then summed for transcripts belonging to the same class. The results (Fig 7A) showed that there was a high degree of heterogeneity in the representation of FREPs between classes and snail strains. For example, FREP A was 2000 fold more highly expressed than FREP E regardless of the snail strain. Between strains, the majority of the transcripts corresponding to FREPs containing one IgSF domain (e.g., FREPs A, D, C1, and C2) were more highly represented in the less-compatible *Bg*BAR strain. Fig 7B shows the summed transcript levels of FREPs containing one or two IgSFs for the four snail strains. The one-IgSF FREPs comprised 80% of the FREP transcripts expressed by *Bg*BAR, but only 55% to 70% of those expressed in the three other snail strains. Moreover, *Bg*BAR was found to globally express more FREPs than the other snail strains (13%, 48% and 52% more than *Bg*GUA, *Bg*VEN, and *Bg*BRE, respectively) (Fig 7B).

### FREP polymorphism analysis

**Fig 8:**
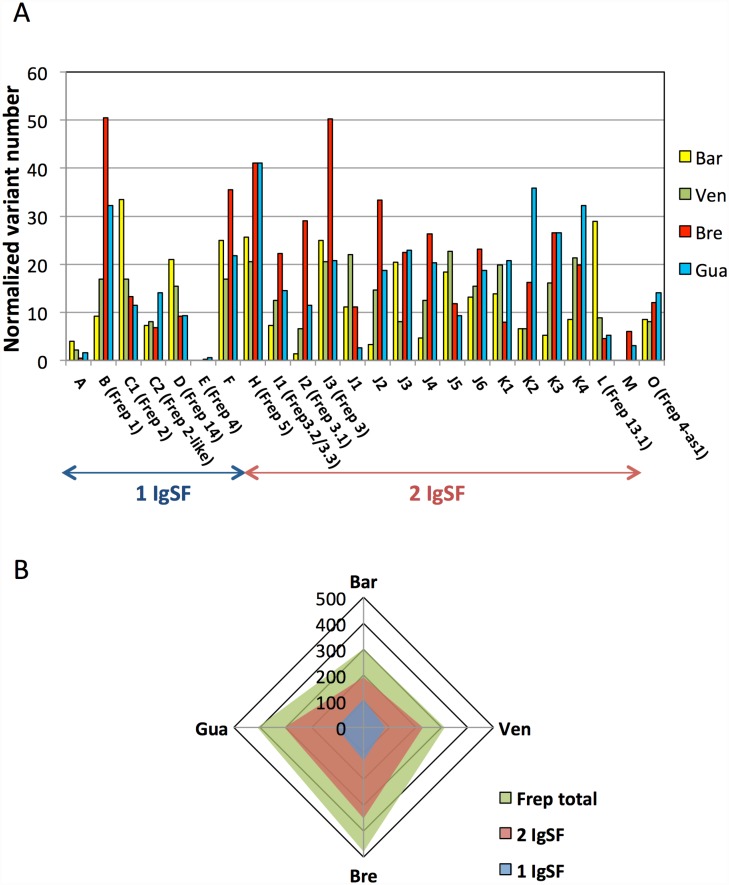
FREP variant polymorphism analysis. Diversity was assessed by aligning the longest transcript from each FREP class to the transcriptomes of *Bg*BAR, *Bg*VEN, *Bg*BRE, and *Bg*GUA using Blastn (similarity, at least 95%). (A) Histograms showing the number of variants normalized by the total number of transcripts for each FREP class in all *B*. *glabrata* strains. (B) For all *B*. *glabrata* strains, diagrams showing the sum of normalized FREP variant numbers based on whether they have one or two IgSF domains.

As most of the recovered FREP transcripts were only partial sequences, we could not align and compare the sequences of the FREP variants. Thus, to estimate their degree of polymorphism, we selected the longest transcript sequence for each of the 24 FREP loci and performed Blastn alignments with each of the *B*. *glabrata* transcriptomes (before CD-Hit EST treatment; Blastn cutoff, 95%). The number of hits was further normalized by the total number of transcripts of each transcriptome. The results (Fig 8A) showed that the variant numbers differed between FREP loci at the intra-strain level. For example, FREPs B and I3 had 50 times more variants than FREP A in *Bg*BRE, while FREP I2 had 30 times fewer variants than FREPs C1 and L in *Bg*BAR. There were also many differences at the inter-strain level; *Bg*BRE (Fig 8B) displayed the highest number of normalized variants (480) followed by *Bg*GUA (409), *Bg*VEN (313), and *Bg*BAR (302). FREP A, E, and M always presented a low number of variants, while FREP F, H, and I3 all had many variants, regardless of the strain (Fig 8A). For the other FREP classes, the numbers of variants differed among the strains, with more variants seen for: FREPs C1 and L in *Bg*BAR; FREPs B, F, I2, I3, and J2 in *Bg*BRE; and FREPs K2 and K4 in *Bg*GUA. For *Bg*VEN, the FREP variants were distributed more or less homogeneously. Notably, although *Bg*BAR had the fewest normalized FREP variants, it displayed the highest numbers of variants for the one-IgSF FREP A, C1 (corresponding to FREP 2 in GenBank), and D (corresponding to FREP 14 in GenBank).

Thus, our results show that *Bg*BAR, which is the least susceptible to *S*. *mansoni* presents the highest level of FREP expression and the lowest number of total FREP variants among the studied strains. Moreover, it expresses a higher proportion of FREPs containing one IgSF domain, and has the highest ratio of one-IgSF variants/all FREP variants.

## Discussion

FREPs and *Sm*PoMucs are key molecular determinants of the compatibility process between *B*. *glabrata* and *S*. *mansoni*. Here, we examined the expression patterns and polymorphisms of these important factors in four host isolates and four parasite isolates that displayed different levels of compatibility with one another. Among the schistosomes, strain *Sm*LE was clearly the most infective against the different snail strains, *Sm*GH2 was the least infective, and the other two strains displayed intermediate compatibility patterns (Fig 1). Among the snails, strain *Bg*BAR was clearly the least permissive, whereas the compatibility phenotypes of the other three strains were much higher and quite similar.

In an effort to explain the compatibility differences among the different schistosome strains, we first analyzed the diversity and expression levels of *Sm*PoMucs in the different strains. The most compatible strains, *Sm*LE and *Sm*VEN, expressed similarly high numbers of *Sm*PoMuc variants (around 270 and 250, respectively; Fig 3), while the two less compatible strains expressed fewer variants (45 and 75 for *Sm*BRE and *Sm*GH2, respectively; Fig 3). These results are clearly in agreement with the hypothesis of a matching phenotype [38]. Indeed, if a population of schistosomes is more diverse in their *Sm*PoMuc patterns, they would be less prone to being recognized by a population of hosts that display a fixed number of FREP immune receptors. These results of *Sm*PoMuc polymorphism analysis clearly agree with the theoretical results obtained from co-evolutionary models applied to plant and animal innate immunity [39]. In the multistep infectious interactions of parasites and hosts, a genetically explicit model revealed that polymorphism will be greater at recognition loci than at effector loci, and that host-genotype by parasite-genotype interactions are greater for the recognition phase than for the effector phase. Our results confirm these predictions, as we herein establish a link between *Sm*PoMuc polymorphism and the compatibility phenotype. Additional results obtained by Nuismer and Dybdahl [39] also showed that, compared to the effector phase, the recognition phase contributes more to local adaptation. This also makes sense in the context of our observation that the compatibility level is generally higher for sympatric combinations. The expression level of *Sm*PoMucs also appears to be relevant to the compatibility pattern. In terms of expression, *Sm*VEN and *Sm*GH2 expressed around two fold more *Sm*PoMuc genes than the other two strains. Conceivably, a schistosome expressing more *Sm*PoMucs will generate more *Sm*PoMuc/FREP interactions at the parasite surface (even given a fixed number of possible combinations), which will favor the recognition, the immune response and the killing of the parasite. This could explain why strain *Sm*VEN expressed a similar number of variants compared with strain *Sm*LE, but displayed a lower compatibility phenotype. Similarly, the expression level of *Sm*PoMucs could explain why *Sm*GH2 was less compatible than *Sm*BRE, even though the former expressed more variants than the latter.

Concerning now the comparison of the proportion of variants belonging to the different groups of *Sm*PoMucs in the studied schistosome strains, it revealed that: (i) the expression level and the number of variants of group 1 *Sm*PoMucs is always low; (ii) the expression levels of groups 2 and 3 *Sm*PoMucs are very similar within each strain; (iii) the proportions of group 2 and 3 variants can differ according to the strain (e.g., group 3 was more diversified in strain *Sm*LE); and (iv) the group 3 of *Sm*PoMucs, which containing intermingled repeats [19,20], are only expressed at significant levels in strain *Sm*GH2. These differences in repeat types or numbers could also explain some of the compatibility differences between strains. However, our current understanding of the link between polymorphism, glycosylation status, and recognition by FREP lectins is too fragmentary to allow us to propose a more precise hypothesis. The *Sm*PoMuc repeat number was shown to be related to the glycosylation status within a specific strain of schistosome [20], but the glycosylation of different variants and its influence on FREP recognition remain to be investigated.

Concerning the FREP contents of the studied snail strains, we first performed a global transcriptomic analysis; we then focused on immune genes, and finally on FREPs. As compatibility depends on molecules that are expressed constitutively by the host and the parasite [18], we conducted our transcriptomic analysis on naive snails. Our global comparative analysis revealed that, as expected, the transcriptome of *Bg*BAR (which was much less permissive than the others (Fig 1) showed the greatest differences with respect to the others. Among the differentially expressed transcripts of this strain, we observed enrichment of numerous genes involved in the immune response and responses to biotic and abiotic stimuli. Accordingly, we performed a more targeted analysis of a *B*. *glabrata*. immunome composed of 122 transcripts encoding proteins known to be involved in the immune recognition, immune signaling pathways, and effector functions of *B*. *glabrata* [17,40]. As expected, *Bg*BAR had a higher proportion of genes that were differentially represented in the majority of these molecular classes and the comparison of the transcription levels of these genes in the different strains showed again that *Bg*BAR is the most important differences. This strain exhibited both over-representation (e.g., FREP1, FREP14, an LBP-BPI 1.1, an achacin, a biomphalysin-like, etc.) and under-representation (e.g., FREP 3.1 M, FREP J1, FREP K2, a biomphalysin-like, a macin, grctm 2, grctm 6, proteases, etc.) of transcripts. As some FREPs were differentially expressed between snail strains, we next analyzed the expression of all FREPs known to be present in the *B*. *glabrata* genome. Our results indicated that *Bg*BAR expressed more FREPs than the three other strains, and that most of the FREPs containing one IgSF domain (5 of 7) were more highly expressed in *Bg*BAR compared to the other strains. This suggests that this specific class of FREPs could play a key role in schistosome recognition and anti-schistosome defense. Indeed, this is consistent with a previous report showing that FREP 2 (containing one IgSF domain) interacts with *Sm*PoMuc antigens [24]. Our analysis of FREP polymorphisms between and within strains showed that *Bg*BRE and *Bg*GUA had more variants than *Bg*VEN and *Bg*BAR, suggesting that the diversity of FREPs is not correlated with the compatibility level, as would be expected from an ongoing arms race between FREPs and *Sm*PoMucs. We thus propose (very speculatively) that this absence of correlation could be a consequence of the recent introduction of the schistosome to the South American and Caribbean areas (from which the tested snail strains were sampled) through the Atlantic slave trade of the 16-19th centuries. This would have forced the schistosome to adapt to a new intermediate host of the same genus (*B*. *pfeifferi* in Africa versus *B*. *glabrata* in the New World). The parasite needed to develop an evolutionary strategy to increase its compatibility with its new host, and thus diversified its *Sm*PoMucs to escape recognition by FREPs. In this context, the diversification of FREPs in some snail strain could be linked to co-evolution with other pathogens living in sympatry with snails of the New World.

Our present results clearly corroborate that FREPs and *Sm*PoMucs seem to be molecular determinants of the compatibility between *B*. *glabrata* and *S*. *mansoni*. However, it is largely accepted that this compatibility process is a complex one that involves numerous genes engaged in an arms race (see [41] for a recent review). Compatibility can be viewed as a multistep process through which the parasite escapes the recognition and effectors of the host. Although FREPs and *Sm*PoMucs may play a crucial part in this recognition, other pattern recognition receptors (e.g., lectins) and antigens have been suggested to be involved [24]. In terms of the effectors of the host and the anti-effector systems of the parasite, it has been shown that highly reactive chemical compounds derived from molecular oxygen (ROS) are crucial to the snail’s ability to defend itself against *S*. *mansoni* [42], and the parasite has developed ROS scavenger systems to counter these molecules [29]. Additional candidate genes have also been recently implicated in the compatibility process between *B*. *glabrata* and *S*. *mansoni*; for example, the biomphalysin from *B*. *glabrata* have been shown to exert high cytotoxicity against *S*. *mansoni* sporocysts [35], and a new family of putative immune receptors was identified using a RAD-seq approach [11]. The previous reports and present results therefore collectively suggest that the compatibility process is likely to be much more complex than previously thought. Indeed, our transcriptomic analysis of *B*. *glabrata* strains showed that the less permissive snail strain varied from the others in terms of immune-relevant transcripts that are involved not only in immune recognition, but also in signaling pathways and effector functions (Fig 6).

In conclusion, the arms race between *B*. *glabrata* and *S*. *mansoni* has selected for diversified molecular repertoires that allow the parasite to counter the immune recognition system of the host. The extraordinary diversification that enables *Sm*PoMucs to avoid recognition by FREPs illustrates the outcome of these evolutionary dynamics. The present findings together with other results obtained in recent decades show that the compatibility between *B*. *glabrata* and *S*. *mansoni* depends on multiple factors, including: (i) the genetics of the snail and the schistosome; (ii) the age of the snail; (iii) the previous interactions of the snail with schistosomes; and (iv) the ability of the environment to influence (through epigenetic mechanisms) the compatible/incompatible phenotypes of both partners (see [41] for a recent review). To solve this complicated puzzle, we need to develop novel integrative approaches that combine comparative genomic, epigenomic, and transcriptomic approaches performed under different environmental conditions. This should enable us to identify relevant candidate genes whose functions could then be validated using CRISPR/Cas or RNAi methodologies. Given the variability of the mechanisms involved in compatibility, such studies must be undertaken on different snail and schistosome populations and strains. These ambitious approaches are absolutely necessary if we hope to identify the molecular pathways that are most likely to be good targets for strategies aimed at blocking transmission through the snail intermediate host.

## Materials and methods

### Ethic statements

Our laboratory holds permit # A66040 for experiments on animals from both the French Ministry of Agriculture and Fisheries, and the French Ministry of National Education, Research, and Technology. The housing, breeding and animal care of the utilized animals followed the ethical requirements of our country. The experimenter also possesses an official certificate for animal experimentation from both French ministries (Decree # 87–848, October 19, 1987). Animal experimentation follows the guidelines of the French CNRS. The different protocols used in this study have been approved by the French veterinary agency from the DRAAF Languedoc-Roussillon (Direction Régionale de l'Alimentation, de l'Agriculture et de la Forêt), Montpellier, France (authorization # 007083).

### Parasites, snails, and compatibility

The four studied strains of *S*. *mansoni* parasites and the four corresponding sympatric snail strains of *B*. *glabrata* originated from South America and had been maintained in the laboratory using Swiss OF1 mice (Charles River Laboratories, France) as the definitive host. The four sympatric host/parasite combinations were designated *Bg*VEN/*Sm*VEN (from Guaraca, Venezuela), *Bg*GUA/*Sm*GH2 (le Lamentin, Guadeloupe), *Bg*BRE/*Sm*BRE (Recife, Brazil), and *Bg*BAR/*Sm*LE (Belo Horizonte, Brazil). Miracidia from the parasite strains (*Sm*BRE, *Sm*LE, *Sm*GH2 and *Sm*VEN) were recovered from infected mouse livers and intestines and transformed into sporocysts in vitro as previously described [19]. Compatibility trials between the strains of parasites and snails were conducted as previously described [43]. For all sympatric and allopatric combinations, we experimentally infected snails with 20 miracidia per snail, which was previously shown to yield a maximum infection rate regardless of the utilized strain [43]. Two weeks later, we assessed the prevalence (percentage of infected snails) and intensity (number of developed parasites per infected snail) of infection. Three independent experiments were performed. The data presented in the present manuscript correspond to the mean values obtained in a previous published work [43] and the data obtained from two other experiments performed in 2010 and 2012.

### Western blot analysis of SmPoMucs in *S*. *mansoni* sporocysts

Five thousand sporocysts were collected and counted for each *S*. *mansoni* strain (*Sm*BRE, *Sm*LE, *Sm*GH2, and *Sm*VEN). The samples were ground with a pestle and vortexed, and proteins were extracted using UTC buffer (7 M urea, 2 M thiourea, 30 mM Tris, pH 8.5, and 4% CHAPS) for 2 h at room temperature. The samples were centrifuged at 10,000 g for 5 min, the supernatants were recovered, and the protein concentrations were estimated using a 2D Quant kit (GE Healthcare Life Sciences). For each sample, proteins (8 μg) were incubated with Laemmli buffer for 5 min at 99°C, resolved by 12% SDS-PAGE, and blotted on a nitrocellulose membrane (Trans-blot Turbo; Bio-Rad). The membrane was blocked with 5% skimmed dry milk in TBST (TBS containing 0.05% Tween 20) for 3 h at room temperature and incubated with the previously described anti-*Sm*PoMuc [24] diluted 1/1000 in TBST overnight at 4°C. The membrane was washed three times in TBST (10 min each, room temperature), and then incubated with peroxidase-conjugated purified anti-rabbit IgG (Sigma Aldrich) diluted 1/5000 in TBST with 5% skimmed dry milk for 90 min at room temperature. The membrane was washed three times in TBST and once in TBS, and proteins were detected with a ChemiDoc MP Imaging system (Bio-Rad) using ECL reagents. As a loading control, we performed a parallel Western blot using anti-actin (Thermo Scientific) diluted 1/1000 and HRP-conjugated anti-mouse IgG (Sigma Aldrich) diluted 1/10,000.

### SmPoMuc polymorphism analysis using individual sporocysts of *S*. *mansoni*

Eleven sporocyts were recovered individually from each *S*. *mansoni* strain. RNA was isolated from each individual using a Dynabeads mRNA Direct Micro kit (Ambion Life Technologies) as previously described [20]. The RNA was reverse transcribed by adding the enzyme mix (Superscript II, Invitrogen) directly to the paramagnetic Dynabeads. The generated cDNA was recovered using the magnetic system, washed twice in 10 mM Tris (pH 7.5) and SmPoMuc sequences were directly amplified using PCR and nested PCR, as previously described [20]. The obtained products were separated by 1% agarose gel electrophoresis. Each band was excised, purified, and cloned into pCR4-TOPO, and 100 clones were sequenced.

### Quantitative PCR analysis of SmPoMuc expression on *S*. *mansoni* sporocysts

Five thousand sporocysts from each *S*. *mansoni* strain were recovered and stored at -80°C. RNA was extracted using a Dynabeads mRNA Direct Micro kit (Ambion Life Technologies) according to the manufacturer’s instructions. Between the washing and elution steps of the RNA purification, an additional on-bead DNase treatment was performed using the TURBO DNA-free kit (Ambion Life Technologies). Reverse transcription was performed using the Maxima H Minus First Strand cDNA Synthesis kit (Thermo Scientific) with a 1:1 mixture of oligo dT and random primers. Quantitative PCR amplifications were performed with 2 μl of 20-fold diluted cDNA and 0.5 μM of each primer in a final volume of 10 μl, using a LightCycler 480 SYBR Green I Master kit and a Light Cycler 480 II Real Time instrument (both from Roche). An initial denaturation at 95°C for 12 min was followed by: 45 cycles of 11 sec denaturation at 95°C, 11 sec annealing at 60°C, and 19 sec elongation at 72°C; a melting curve step from 65 to 97°C with a heating rate of 0.11°C/sec and continuous fluorescence measurement; and a cooling step to 40°C. For each reaction, the cycle threshold (Ct) was determined using the 2nd derivative method of the LightCycler 480 Software release 1.5 (Roche). PCR experiments were performed in triplicate (technical replicates) from three biological replicates. The mean value of Ct was calculated. Corrected melting curves were checked using the Tm-calling method of the LightCycler 480 Software release 1.5. Results were normalized with respect to the α-tubulin gene, as previously described [22], and ΔCt values were calculated. The primers used to analyze the different *Sm*PoMuc groups are indicated in Fig 1A.

### *B*. *glabrata* cDNA library construction and sequencing

RNA extraction, cDNA library construction, and Illumina SOLEXA sequencing were performed as previously described [33,44]. Briefly, total RNA was extracted from whole snail body tissues from 10 juvenile, 10 adult, and 10 old snails for each *B*. *glabrata* strain (*Bg*VEN, *Bg*GUA, *Bg*BRE, and *Bg*BAR). Tissues were disrupted in liquid nitrogen and total RNA was extracted using the TRIzol reagent (Life Technologies) according to the manufacturer’s instructions. Equimolar amounts of RNA from juvenile, adult and old *B*. *glabrata* were combined to yield two pools of 30 individuals for *Bg*BRE (BRE1 and BRE2) and one pool of 30 individuals for each of the other strains (*Bg*BAR, *Bg*VEN and *Bg*GUA).

Paired-end 72-bp cDNA libraries were generated using an mRNA-seq kit for transcriptome sequencing (Solexa, Illumina) on a Genome analyzer II platform (Illumina). Three samples were multiplexed per lane. Library construction and sequencing were performed by MGX (Montpellier Genomix, c/o Institut de Génomique Fonctionnelle, Montpellier, France). For library constructions cDNA fragments following RT-PCR amplification ranged from 220 to 500 bp (average, 300 bp). The numbers of 72-bp reads obtained from *Bg*BRE1, *Bg*BRE2, *Bg*BAR, *Bg*GUA, and *Bg*VEN were 99,316,948, 73,000,210, 116,679,444, 100,640,190 and 111,178,786, respectively. The reads that passed the Illumina quality filter were further cleaned via a previously described workflow, using the Galaxy server [33,44]. The 13 first and three last low-quality bases were then trimmed; yielding paired-end reads of 56 nucleotides each. Reads without a pair (orphan reads) were removed. The final high-quality libraries used for transcriptome assemblies contained 90,331,578, 66,558 544, 105,718,404, 88,800,366, and 85,918,982 reads for *Bg*BRE1, *Bg*BRE2, *Bg*BAR, *Bg*GUA, and *Bg*VEN, respectively.

### Processing of read sequences obtained from RNA-seq of *B*. *glabrata*

*De novo* transcriptomic assemblies were performed using the Velvet version 1.2.02 software implemented by the python script provided by Oases version 0.2.06, as previously described [33,44]. The resulting contigs were merged into unigene clusters using CD-HIT-EST version 4.5.4 A multiple k-mer assembly approach was applied to optimize the assembly. Four high-quality reference transcriptomes were produced, comprising 117,269, 70,533, 82,500, and 79,664 transcripts for strains *Bg*BRE, *Bg*BAR, *Bg*GUA, and *Bg*VEN, respectively (for transcriptome details see S4 Table). The transcripts were automatically annotated using Blast2GO version 2.4.2.

### Differential expression of transcripts in *B*. *glabrata*

Quality reads (Phred score >26) were aligned on the *Bg*BRE transcriptome assembly using Bowtie2 (v2.0.2; mapping quality score 255) on the Galaxy server (http://bioinfo.univ-perp.fr) [45]. The count of the reads mapped to each transcript was assessed using Hitcount to BAM (SAM tools v0.1.18.0). The obtained values were normalized by the upper quartile method [46], which has been proposed to be more accurate than the RPKM method of normalization [47]. The DESeq2 software (v2.12; http://www.bioconductor.org/packages/release/bioc/html/DESeq2.html) [48] was used under default settings to identify genes that were differentially expressed in the two biological snail sample duplicates (BRE1 and BRE2) versus the other snail strain samples (P<0.05). Hierarchical ascending clustering (HAC) with Pearson correlation, which was performed using the Cluster 3.0 [49] and JavaTreeView software packages, was used to generate a heatmap for analysis of the transcript expression patterns (log2 fold change).

### Gene Ontology (GO) enrichment analysis

To detect biological processes that were significantly over- or under-represented in the *Bg*BAR strain, we examined the transcripts found to be differentially expressed between *Bg*BAR and *Bg*BRE. Functional enrichment was assessed using the GoStatsPlus package in R (https://github.com/davfre/GOstatsPlus). The enrichment was calculated for the 3 categories including of biological process (BP), molecular function (MF), and cellular component (CC), and. The results were imported into REVIGO [50] for further analysis and visualization.

### Sequence analysis of *B*. *glabrata* FREPs

Transcripts corresponding to potential FREP sequences were selected from transcriptomes on the basis of Blast2Go annotations or the presence of immunoglobulin and/or fibrinogen domains (IPR013783 and IPR002181, respectively), as detected by Interproscan. As most of the obtained sequences were not full length, transcripts encoding complete sequences or partial sequences with contiguous Immunoglobulin superfamily (IgSF) and fibrinogen (FBG) domains were selected to be sure to have a transcript belonging to a real FREP family member and not to GREP, CREP, FREM or IgSF or FBG containing molecules. The retained transcripts were translated to predicted amino acid sequences and aligned with those of reference FREP proteins obtained from GenBank using BioEdit version 7.1.3.0. and were also aligned on the *B*. *glabrata* genome draft using the Blast tool available on the Vector Base website (https://www.vectorbase.org/). For quantitative analysis of FREPs, the reads from each *B*. *glabrata* strain were mapped to a reference FREP transcriptome (FREP transcripts assembled from the transcriptomes of the four snail strains) using Bowtie2 (v2.0.2). The mapped reads were counted per transcript and the values were normalized by the upper quartile method, as described above. To estimate the FREP polymorphism in each strain, the longest transcript from each FREP class was aligned against the *B*. *glabrata* transcriptomes obtained before CD-Hit EST treatment, using Blastn (cutoff, 95% identity) on the Galaxy server. The number of mapped hits was further normalized to the total number of transcripts for each *B*. *glabrata* transcriptome, and the level of polymorphism was calculated as follows for each FREP class and snail strain: normalized number of FREP variants = (number of blast hits / total number of transcripts) x 10^5^.

### Validation of *de novo* transcript assembly

Several d*e novo* assembled transcripts were validated by traditional Sanger sequencing of PCR products. Briefly, depending of the strain, total RNA was reverse transcribed with random primers and RevertAid premium enzyme (Thermo scientific). Two μl of the RT reaction was then used for PCR (Advantage 2 PCR system, Invitrogen, Carlsbad, CA, USA) with primers that were designed to specially target and amplify novel predicted transcripts, and amplicons were sequenced (GATC Biotech, Konstanz, Germany). Sequences from Sanger sequencing and from computational assembly of *de novo* transcripts were aligned (Supplementary S1 Fig).

### Accession numbers

The obtained *Sm*PoMuc sequences were deposited in GenBank under the following accession numbers: KX645102-KX645377 for *Sm*LE, KX645378-KX645624 for *Sm*VEN, EU676447-EU676459/EU676503-EU676530/EU676572-EU676583/EU676595-EU676625 for *Sm*BRE, and EU676460-EU676502/EU676531-EU676571/EU676584-EU676594/EU676556-EU676626 for *Sm*GH2.

The obtained FREP sequences were deposited in GenBank under the following accession numbers: KY024239 to KY024307.

## Supporting information

S1 Fig Validation of FREP by alignment of sequences obtained from Sanger sequencing and from computational assembly of *de novo* transcripts. To confirm that *de novo* assembly will not generate miss assemblies for highly diverse FREP molecules, assembled FREP transcripts were validated by traditional Sanger sequencing of PCR products. Sanger sequences and computational assembled transcripts were aligned. Nucleotides underlined in black color are common to both sequences. Forward and reverse primers positions were indicated by asterisks.
(PDF)

S1 Table Variants of cDNA obtained from 11 individual sporocysts of *S*. *mansoni* strains. (XLS)

S2 Table *B glabrata* immunome. List of molecule categories previously shown to play roles in snail immunity, and the corresponding loci in the *Bg*BRE transcriptome. Underlined lanes correspond to transcripts that are differentially expressed across the four *B*. *glabrata* strains. Molecules were selected from: *B*. *glabrata* transcriptomes and the literature [11,17,30]. FREP nomenclature is detailed in the chapter “Characterization and expression analysis of FREPs in the four *B*. *glabrata* strains”, see Results section.
(XLSX)

S3 Table Annotation of the 24 classes of FREP molecules identified. The longest transcript number of each class is mentioned, as are: its length in nucleotides and in amino acids (after virtual translation); its new FREP class and standard classification when possible; its IgSF domain number; its position on the *B*. *glabrata* genome draft (genome assembly version BglaB1); the corresponding exon number; and, when determined, the predicted transcript in the Vectorbase database.
(XLSX)

S4 Table Transcriptome statistics. The characteristics of *Bg*BAR, *Bg*BRE1, *Bg*BRE2, *Bg*VEN and *Bg*GUA transcriptomes were detailed in terms of reads, contig length, contig number and nucleotide numbers. *Bg*BRE1 and *Bg*BRE2 transcriptomes came from 2 independent biological replicates.
(XLSX)
