# Supplementary material for: A multistrain approach to studying the mechanisms underlying compatibility in the interaction between Biomphalaria glabrata and Schistosoma mansoni
Source: PLoS Negl Trop Dis. 2017 Mar 2;11(3):e0005398. doi: 10.1371/journal.pntd.0005398 (PMC5349689; doi:10.1371/journal.pntd.0005398)
Supplement: S1 Fig — To confirm that de novo assembly will not generate miss assemblies for highly diverse FREP molecules, assembled FREP transcripts were validated by traditional Sanger sequencing of PCR products. Sanger sequences and computational assembled transcripts were aligned. Nucleotides underlined in black color are common to both sequences. Forward and reverse primers positions were indicated by asterisks. (PDF) [file pntd.0005398.s001.pdf]

FREP A

```
                                       510       520       530       540       550       560       570       580       590       600
                                ....|....|....|....|....|....|....|....|....|....|....|....|....|....|....|....|....|....|....|....|
FREP_A_Locus_1043_Tr_1          CCTCCACGGAAGAATACGTCTCGACACTGTTTCAGCTTCACAAGGAGCTGGATCAGAGCCAGGCAAAGCTGAATGCGGCTTATGCTGCTGGAAACAAGAC
PCR sequence from FREP_A        ----------------------------------------------------------------------------------------------------
                                                                                      ************************
                                       610       620       630       640       650       660       670       680       690       700
                                ....|....|....|....|....|....|....|....|....|....|....|....|....|....|....|....|....|....|....|....|
FREP_A_Locus_1043_Tr_1          ACAGTCGGAGCTCGTGGAAACCCAGACAAGACTTTCTAGACGTCAGTTACCAGACGACTGCATCCCTGTTGTTTCCAACGCGACAGGATGCGATGACCCC
PCR sequence from FREP_A        -------------------------------CTTTCTAGACGTCAGTTACCAGACGACTGCATCCCTGTTGTTTCCAACGCGACAGGATGCGATGACCCC

                                       710       720       730       740       750       760       770       780       790       800
                                ....|....|....|....|....|....|....|....|....|....|....|....|....|....|....|....|....|....|....|....|
FREP_A_Locus_1043_Tr_1          AAGTTGACCGTCGGCTACCACATCCTGGAAATCGTCCCCAAAGACGGGCTGGGTCCCATCAAAGTTTTATGCCACGTTCGCATCCAAGGTGAAGGCTGGA
PCR sequence from FREP_A        AAGTTGACCGTCGGCTACCACATCCTGGAAATCGTCCCCAAAGACGGGCTGGGTCCCATCAAAGTTTTATGCCACGTTCGCATCCAAGGTGAAGGCTGGA

                                       810       820       830       840       850       860       870       880       890       900
                                ....|....|....|....|....|....|....|....|....|....|....|....|....|....|....|....|....|....|....|....|
FREP_A_Locus_1043_Tr_1          TCGTGTTCCAGAAGAGATTCGACGGCTCTGTCGACTTCTACAGAAATTTCCGCGATTACGAAGTCGGGTTCGGCACAGTTAGTCCGACCACAGAGTTCTG
PCR sequence from FREP_A        TCGTGTTCCAGAAGAGATTCGACGGCTCTGTCGACTTCTACAGAAATTTCCGCGATTACGAAGTCGGGTTCGGCACAGTTAGTCCGACCACAGAGTTCTG

                                       910       920       930       940       950       960       970       980       990      1000
                                ....|....|....|....|....|....|....|....|....|....|....|....|....|....|....|....|....|....|....|....|
FREP_A_Locus_1043_Tr_1          GCTGGGTTTGGAGAACCTCAGACGTCTCCTTGTTGACAACGGTGACGAGAACGGGCTTCGCGTGGACATGACAGAAAGAGGCACTGCCTTCAACTTCACC
PCR sequence from FREP_A        GCTGGGTTTGGAGAACCTCAGACGTCTCCTTGTGGACAACGGTGACGAGAACGGGCTTCGCGTGGACATGACAGAAAGAGGCACTGCCTTCAACTTCACC

                                      1010      1020      1030      1040      1050      1060      1070      1080      1090      1100
                                ....|....|....|....|....|....|....|....|....|....|....|....|....|....|....|....|....|....|....|....|
FREP_A_Locus_1043_Tr_1          AGAATATACCCCATTTTCCGAGTAGGTCCAGGAGACGGCTACACCCTGATCGCTGACGGATACAGCGACGGAGGCAAGGGTCTGTCCGAGAACTCTGGCG
PCR sequence from FREP_A        AGAATATACCCCATTTTCCGAGTAGGTCCAGGAGACGGCTACACCCTGATCGCTGACGGATACAGCGACGGAGGCAAGGGTCTGTCCGAGAACTCTGGCG

                                      1110      1120      1130      1140      1150      1160      1170      1180      1190      1200
                                ....|....|....|....|....|....|....|....|....|....|....|....|....|....|....|....|....|....|....|....|
FREP_A_Locus_1043_Tr_1          CCAAGTTCTCCACCTACGACAACGATCACTCCCTTGGGTGCCCTTCAAGTCTGCGTCTCGCTGGTTGGTGGTTCCACGAAGGCTGTGGCTTCGTCAATCT
PCR sequence from FREP_A        CCAAGTTCTCCACCTACGACAACGATCACTCCCTTGGGTGCCCTTCAAGTCTGCGTCTC-----------------------------------------
                                                                           **********************
```

FREP D-as1

```
                                       310       320       330       340       350       360       370       380       390       400
                                ....|....|....|....|....|....|....|....|....|....|....|....|....|....|....|....|....|....|....|....|
FREP_D-as1_Locus_15883_Tr_6     TGCCCATGGGAAAATCTATAACAAAGACTTGCAAGACTCTTACCTTCAAGTAATTTGGAAGAATCCGAAGATCTCCGAGTCTGGAAAATATTTCTGCCTA
PCR sequence from FREP_D-as-1   ----------------------------------------------------------------------------------------------------
                                                                                               ********************
                                       410       420       430       440       450       460       470       480       490       500
                                ....|....|....|....|....|....|....|....|....|....|....|....|....|....|....|....|....|....|....|....|
FREP_D-as1_Locus_15883_Tr_6     GCATACGCCAAGAATTCTACTGGCCAAGATAGTGTGTTTCAATCCACAGTAACAATAAAAGTTTTAAAACCAAAGGCGGATGATTTGGTCCAGGTGTTAG
PCR sequence from FREP_D-as-1   ----------AGAATTCTACTGGCCAAGATAGTGTGTTTCAATCCACAGTAACAATAAAAGTTTTAAAACCAAAGGCGGATGATTTGGTCCAGGTGTTAG

                                       510       520       530       540       550       560       570       580       590       600
                                ....|....|....|....|....|....|....|....|....|....|....|....|....|....|....|....|....|....|....|....|
FREP_D-as1_Locus_15883_Tr_6     GCCAGCTACTTAAGAGAGTAGATACACTTGAACAACTACTGAAAGGAAACGAAACAAATTTTGGAGGTGTGACAGCAGACGACATATTGATTTCAACAAC
PCR sequence from FREP_D-as-1   GCCAGCTACTTAAGAGAGTAGATACACTTGAACNNCTACTGAAAGGAAACGAAACAAATTTTGGAGGTGTGACAGCAGACGACATATTGATTTCAACAAC
```

```
                                          610       620       630       640       650       660       670       680       690       700
                                  ....|....|....|....|....|....|....|....|....|....|....|....|....|....|....|....|....|....|....|....|
FREP_D-as1_Locus_15883_Tr_6       CCATAAAACACCGTTCACTTCCTGTCGTGACGTCAACAGCTCAGATGAACGCGTGGTGGTGACTTTAGCTTCAGAGCAAAAGGTCATGTGTGACACCAAG
PCR sequence from FREP_D-as-1     CCATAAAACACCGTTCACTTCCTGTCGTGACGTCAACAGCTCAGATGAACGCGTGGTGGTAACTTTAGCTTCAGAGCAAAAGGTCATGTGTGACACAAAG

                                          710       720       730       740       750       760       770       780       790       800
                                  ....|....|....|....|....|....|....|....|....|....|....|....|....|....|....|....|....|....|....|....|
FREP_D-as1_Locus_15883_Tr_6       ACAGACGGCGGTGGATGGATCATCTTTCAGAGAAGAATTATGGGATATGTTGATTTCTACAGAGGCTGGAAGGAGTATCGTGACGGCTTTGGTGATTACG
PCR sequence from FREP_D-as-1     ACAGATGGCGGCGGTTGGATTATCATCCAA----------------------------------------------------------------------
                                                                                                                ********************

                                          810       820
                                  ....|....|....|....|....|.
FREP_D-as1_Locus_15883_Tr_6       ACATTGGTGAATTTTATCTGGGAAAT
PCR sequence from FREP_D-as-1     --------------------------
                                  ***********
```

FREP I2

```
                                          510       520       530       540       550       560       570       580       590       600
                                  ....|....|....|....|....|....|....|....|....|....|....|....|....|....|....|....|....|....|....|....|
FREP_I2_Locus_3534_Tr_70          GTTAACAATAGAAGTTATAAGTCCAACAATTGATGATTTGATGGAAGTAATCCAAAAATTAGTTACACAGGTTGATGGAGACAAGGAAAGCCTGCAAGAT
PCR sequence from FREP I2         --------------------------------------------------------------------------------------AAAGCCTGCAAGAT
                                                                                   *************************

                                          610       620       630       640       650       660       670       680       690       700
                                  ....|....|....|....|....|....|....|....|....|....|....|....|....|....|....|....|....|....|....|....|
FREP_I2_Locus_3534_Tr_70          GTTAAACAAAATATAAAGAACATTAAAGAAGACTTAAACACTAAAGAACAAAATATTATTAGCATTAAAGAGGATTTGAATACTAAACAACAAAGTATCA
PCR sequence from FREP I2         GTTAAACAAAATATAAAGAACATCAAAGAAGACTTGGATTCTAAAGAACAAAATATCATTAGCATTAAAGAGGATGTGATTTACAAATCAACATAGTATCA

                                          710       720       730       740       750       760       770       780       790       800
                                  ....|....|....|....|....|....|....|....|....|....|....|....|....|....|....|....|....|....|....|....|
FREP_I2_Locus_3534_Tr_70          TAAGCATTAAAGAAGAATTCAAAACTAAACAAGAAACTATTCAAAAAGACGTGACTATAAATCAACAAAATATACAGAAAATTAAAGAAGAATTGGATTC
PCR sequence from FREP I2         TAATCCTTAATGAAGACTTCAAAATTAAACAAGAAACTATTCAAAAAGACGTGACTATAAATCAACAAAATATACAGAAAATTAAAGAAGAATTGGATTC

                                          810       820       830       840       850       860       870       880       890       900
                                  ....|....|....|....|....|....|....|....|....|....|....|....|....|....|....|....|....|....|....|....|
FREP_I2_Locus_3534_Tr_70          TAAAGAACAAAGCATGATAAGGATTAGAGAAGACTTCAGCGCGTGTCAACAGAATATAAGCGCATTTAAAGAAAATCTCGAAATAATGTTTGTTAATCTT
PCR sequence from FREP I2         TAAAGAACAAAGCATGATAAGGATTAGAGAAGACTTCAGCGCGTGTCAACAGAATATAAGCGCATTTAAAGAAAATCTCGAAATAATGTTTGTTAATCTT

                                          910       920       930       940       950       960       970       980       990      1000
                                  ....|....|....|....|....|....|....|....|....|....|....|....|....|....|....|....|....|....|....|....|
FREP_I2_Locus_3534_Tr_70          TCAACTTCTTTCATGCAAGTGAAGACACAAATTAATAAAGGAAACCAAGAAGACCAACATTTAATACCCCAGAAGATTTGTCGGGACGTCAACAGTACCG
PCR sequence from FREP I2         TCCACTTCTTTCATGCAAGTGAATACACAAATTAATAAAGGAAACCAAGAAGACCAACATTTAATACCCCAGAAGATTTGTCGGCACGTCAACAGTACCG

                                         1010      1020      1030      1040      1050      1060
                                  ....|....|....|....|....|....|....|....|....|....|....|....|
FREP_I2_Locus_3534_Tr_70          ACGAGCGTGTGGTGGTAACATTAACTTCCGGGTTAAAGGTCATGTGTGACACCAAGACAG
PCR sequence from FREP I2         A-----------------------------------------------------------
                                                        **********************
```

FREP K3

```
                                   510       520       530       540       550       560       570       580       590       600
                            ....|....|....|....|....|....|....|....|....|....|....|....|....|....|....|....|....|....|....|....|
FREP_K3_Locus_3534_Tr_154   TCTGAGGTCAATCAAAAATCAAGATTTCAATTTTACGGAAGTTCTGACATTATTCAAGAACTAATTGAGCCTTTGACATTAAAGTGTTCATATAAGACTT
PCR sequence from FREP_K3   ----------------------------------------------------------------------------------------------------
                                                                                    ***********************
                                   610       620       630       640       650       660       670       680       690       700
                            ....|....|....|....|....|....|....|....|....|....|....|....|....|....|....|....|....|....|....|....|
FREP_K3_Locus_3534_Tr_154   TAAGCCAAGGTCATAAGGAAACTTCTACTATTCAATCTCTGTTTATACTTCATGAGACTAATGGTGTCATTGCATATATAAATAAACATCAACCCGTTGT
PCR sequence from FREP_K3   -------AGGTCATAATGAATCGTCTACTATTCAATCTTTGTTTATACTTCATGAGACTAATGGTGTCATTGCATATATAAATAAACATCAACCCGTTGT

                                   710       720       730       740       750       760       770       780       790       800
                            ....|....|....|....|....|....|....|....|....|....|....|....|....|....|....|....|....|....|....|....|
FREP_K3_Locus_3534_Tr_154   TACAACCATTCAAGGAGATAATTTAAAAAATGTTGAAGGTGAAATCTTTGACAACGAATCAAAAGATTCCTATCTTCAAGTCACGTGGAACAATTTGAAA
PCR sequence from FREP_K3   TACAACCATTCAAGAAAGAAATTTAAAAAATGTTGAAGGTGAAATCTTTGACAACGAATCAAAAGATTCCTATCTTCAAGTCACATGGAACTATTTGAAA

                                   810       820       830       840       850       860       870       880       890       900
                            ....|....|....|....|....|....|....|....|....|....|....|....|....|....|....|....|....|....|....|....|
FREP_K3_Locus_3534_Tr_154   CATTCAGAATCTGGAAAATATTTTTGTGAAGCTCATGTCAACTGTTCAGATGGAAGATTTGATAAGCTCATTGACATTGTAACAATAATAGTACGAGGTT
PCR sequence from FREP_K3   CATTCAGAATCTGGAAACTATTTTTGTGAAACTCATGTCATTCATTCAGATGGAAAATTTGATAACATAAATGAATTGTTAACAATTACAGTACAAAGTC

                                   910       920       930       940       950       960       970       980       990      1000
                            ....|....|....|....|....|....|....|....|....|....|....|....|....|....|....|....|....|....|....|....|
FREP_K3_Locus_3534_Tr_154   CAACACTAGAGGATTTGGTGAAAGTAATACAGAAATTACAAAGACAGGCTGAAGTAGACCAAGAAAGCCTACAAAATAATGAACAAAAAATAAAGGCCAT
PCR sequence from FREP_K3   CACCACTTGATGATTTGGTGAAATTAATACAGAACTTACAAAGACTTGCTGAAGTAAACAAAGAAGC---------------------------------
                                                                                 ************************
```

FREP M

```
                                   710       720       730       740       750       760       770       780       790       800
                            ....|....|....|....|....|....|....|....|....|....|....|....|....|....|....|....|....|....|....|....|
FREP_M_Locus_70165_Tr_6     GATCAAGACGCGAAAGACTACCAAGAACCATTAAAACTTGTAAGCGGAAACCTTAACGGTGGAAACCTCAGTGAACCCAGTCTTCAAGTCACTTGGACCA
PCR sequence from FREP_M    -------------------------------------------------------------GAAACCTCAGTGAACCCAGTCTTCAAGTCACTTGGACCA
                                  **********************
                                   810       820       830       840       850       860       870       880       890       900
                            ....|....|....|....|....|....|....|....|....|....|....|....|....|....|....|....|....|....|....|....|
FREP_M_Locus_70165_Tr_6     AACCAAGTTTTTCACTCTCTGGAAACTATTTCTGTGGAGCACATACTGTGAATAACAGAGGGGATGAGAGAAGATATAACGCGAGACTTACAGTGACTGT
PCR sequence from FREP_M    AACCAAGTTTTTCACTCTCTGGAAACTATTTCTGTGGAGCACATACTGTGAATAACAGAGGGGATGAGAGAAGATATAACGCGAGACTTACAGTGACTGT

                                   910       920       930       940       950       960       970       980       990      1000
                            ....|....|....|....|....|....|....|....|....|....|....|....|....|....|....|....|....|....|....|....|
FREP_M_Locus_70165_Tr_6     TGAAGAGACAAAGCTGGAAGATCTAGTTAGTATTGTGAGTGATTTGCGACAACAAGTTAATGAGTTCCAAGAAGAACAGGAGACGAACAAAGGAAGAGAA
PCR sequence from FREP_M    TGAAGAGACAAAGCTGGAAGATCTAGTTAGTATTGTGAGTGATTTGCGACAACAAGTTAATGAGTTCCAAGAAGAACAGGAGACGAACAAAGGAAGAGAA

                                  1010      1020      1030      1040      1050      1060      1070      1080      1090      1100
                            ....|....|....|....|....|....|....|....|....|....|....|....|....|....|....|....|....|....|....|....|
FREP_M_Locus_70165_Tr_6     ATTAACTCTGCTCTGAGCATAGAAAGAGTTATCGATAATTTAACTTCTGTTGAAACATACTGGAAAAAGAGATTTGATGAAGATATTCTTCCTATGAAAA
PCR sequence from FREP_M    ATTAACTCTGCTCTGAGCATAGAAAGAGTTATCGATAATTTAACTTCTGTTGAAACATACTGGAAAAAGAGATTTGATGAAGATATTCTTCCTATGAAAA

                                  1110      1120      1130      1140      1150      1160      1170      1180      1190      1200
                            ....|....|....|....|....|....|....|....|....|....|....|....|....|....|....|....|....|....|....|....|
FREP_M_Locus_70165_Tr_6     ACGAAATAAGGAAGATTATTGCTGATCAAGAAATGATGGATATAGCTCCATATCTATGTCATGATGTTGAAAGTACTGAAGACCGCGTTGTGGTAACTTT
PCR sequence from FREP_M    ACGAAATAAGGAAGATTATTGCTGATCAAGAAATGATGGATATAGCTCCATATCTATGTCATGATGTTGAAAGTACTGAAGACCGCGTTGTGGTAACTTT
```

```
                                   1210      1220      1230      1240      1250      1260      1270      1280      1290      1300
                          ....|....|....|....|....|....|....|....|....|....|....|....|....|....|....|....|....|....|....|....|
FREP_M_Locus_70165_Tr_6   ACCTTCAGGGTTAAAGGTTATGTGTGACACCAAGACAGAAGGTGGTGGGTGGATCATCATTCAGAGAAGAATCAACGGAAATGTTGATTTCTACAGAAAT
PCR sequence from FREP_M  ACCTTCAGGGTTAAAGGTTATGTGTGACACCAAGACAGAAGGTGGTGGGTGGATCATCATTCAGAGAAGAATCAACGGAAAAGTAGATTTCTATAGAAGC

                                   1310      1320      1330      1340      1350      1360      1370      1380      1390      1400
                          ....|....|....|....|....|....|....|....|....|....|....|....|....|....|....|....|....|....|....|....|
FREP_M_Locus_70165_Tr_6   TGGCAGGCATACCGTGACGGCTTTGGTGATTTCAACATTGGTGAATTTTACCTGGGAAATGAAATTATTTTTAAATTGACCACTAAAAGAAAATATAAAC
PCR sequence from FREP_M  TGGAAGGAGTATCGTGACGGCTTTGGAGATTTCAACATTGGTGAATTTTACCTGGGAAATGAAATTATTTTTAAATTGACCACTAAAAGAAAATATAAAC

                                   1410      1420      1430      1440      1450      1460      1470      1480      1490      1500
                          ....|....|....|....|....|....|....|....|....|....|....|....|....|....|....|....|....|....|....|....|
FREP_M_Locus_70165_Tr_6   TAAGAATCGATTTGAAATATGGAAACACGACATATTTTGCACAATACTCAGATTTTAAATTGTTAGGTGAAAACAACAAGTTTAAACTCAAAATAGGAAA
PCR sequence from FREP_M  TAAGAATCGATTTGAAATATGGAAACACGACATATTTTGCACAATACTCAGATTTTAAATTGTTAGGTGAAAACAACAAGTTTAAACTCAAAATAGGAAA

                                   1510      1520      1530      1540      1550      1560      1570      1580      1590      1600
                          ....|....|....|....|....|....|....|....|....|....|....|....|....|....|....|....|....|....|....|....|
FREP_M_Locus_70165_Tr_6   TTATTCAGGTAACGCTGGCGATAGTCTAAAACGACATCATAATAAATATTTCACAACATTCGATAAAAACAACGATAGGACAGGCTGGCATTGCGCTAAA
PCR sequence from FREP_M  TTATTCAGGTAACGCTGGCGATAGTCTAAAACGACATCATAATAAATATTTCACAACATTCGATAAAAACAACGATAGGACAGGCTGGCATTGCGCTAAA
                                                                                                                  ************
                                   1610      1620      1630      1640      1650      1660      1670      1680      1690      1700
                          ....|....|....|....|....|....|....|....|....|....|....|....|....|....|....|....|....|....|....|....|
FREP_M_Locus_70165_Tr_6   ACCAGGCACGGTGCCTGGTGGTACGATGGATGTTCTGATTCAAACCTTAACGGCAATTGGGGAAGAGCCGAACCCGATGGCCTCTACTGGGATACTATAG
PCR sequence from FREP_M  ACCAGGCA--------------------------------------------------------------------------------------------
                          ********
```
